# Music and neuroscience research for mental health, cognition, and development: Ways forward

**DOI:** 10.3389/fpsyg.2022.976883

**Published:** 2022-08-25

**Authors:** Maria Agapaki, Elizabeth A. Pinkerton, Efthymios Papatzikis

**Affiliations:** ^1^Department of Early Childhood Education and Care, Oslo Metropolitan University, Oslo, Norway; ^2^Independent Researcher, Dubai, United Arab Emirates

**Keywords:** mental health, cognition, music, neuroplasticity, research quality, development, music neuroimaging

## Introduction

The brain function on music has been a recently developing field of neuroscience that holds a position of great interest to neuroscientists, psychologists, health professionals, and musicians alike. The primary goal of investigating music and the brain has been to determine particular neural correlates that are involved in or altered by the engagement of humans with music. It can be said that the study of the neuroscience behind music is a discussion regarding human behavior, environmental stimuli, and how that can be represented in our physiology, as well as how our brain structure allows us to interact with such stimuli in a unique and functional way.

Music is a complex phenomenon that employs, from very early in life, widespread neural activity in interconnected regions of sensory perception (Papatzikis et al., 2019), and ranging from the auditory cortex (Brattico et al., 2006; Allen et al., 2017) to the motor system (Furukawa et al., 2017; Bashwiner and Bacon, 2019) during active and passive music listening or instrument training. By the same token, the music's impact has been greatly correlated to both ontogenetic and phylogenetic neuroplastic changes (Papatzikis and Rishoni, 2022), showcasing a strong link to human brain function proven through a plethora of neuroimaging modalities [please see for example (Lin et al., 2010; Cross and Fujioka, 2019) for EEG/ERP studies; (Donnay et al., 2014) for fMRI; (Chacon-Castano et al., 2017) for MEG; (Moore et al., 2014) for DTI; (Sluming et al., 2002) for Voxel-based Morphometry]. However, emerging data suggest that the association between music and the brain is markedly more intricate than simply the response to sensory stimuli. For instance, music has been implicated in contexts of emotional, social, cultural, and biological influence (Peretz, 2006; Koelsch, 2018; Savage, 2019; Savage et al., 2020). Developmental neuroscience has studied the processing and perception of music in the fetal and infant brain and its selective role in environmental enrichment and socioemotional development (Papatzikis and Papatziki, 2016; Chorna et al., 2019; Arrasmith, 2020; Papatzikis et al., 2021). Mental health research suggests the potential benefits of music in alleviating symptoms in a variety of neurological and affective disorders ranging from depression and schizophrenia to dementia (Van de Winckel et al., 2004; Talwar et al., 2006; Lin et al., 2011; Gustavson et al., 2021). Cognitive studies of attention have even observed executive system efficiency differences when comparing the attentional network test (ANT) scores of the alerting and orienting networks of musicians and non-musicians (Medina and Barraza, 2019).

Considering music research encompasses a plethora of fields in psychology and neuroscience, and that the current advancements in neuroimaging technologies have made research questions of interest in the field substantially more feasible and diversified, our investigations require a sufficient foundation of quality and validity that ensures the field to move in an effective progression. The assessment of the primary qualitative and quantitative studies that drive the field forward is a necessary systematic review to acknowledge and evade issues like bias, inadequate methodology, or a reproducibility crisis. Previous exemplary studies of the analysis of research quality in other various fields have summarized challenges and subsequent directions, as in the field of population neuroscience (Paus, 2010), or have even provided guidelines to address future studies, as in the field of the neuroscience of information systems (Brocke and Liang, 2014). Both approaches advocate such consideration to obtain and maximize the potential of neuroscience research.

Therefore, the quality and logistics of research are significant factors that must be adequately regulated to set a standardized precedent for future experimentation within the field. Without doing so, research in music and neuroscience enables the risk of error, bias, and deficient methodology which, in turn, impedes the progression of the field. For instance, in the field of behavioral neuroscience, Bespalov and Steckler (2018) suggest a current lack of quality control sparked by criticisms regarding poor design, misreporting, and lack of power. Recognizing that the alternative for inadequate research quality would be that which is credible and valuable, it can be implied there are two possible directions for the neuroscience of music. What current research indicates might give us an understanding of which direction that would be, as well as what to do to avoid such devaluation, further justifying the importance of such quality studies.

## The current debate

A great and controversial discussion referring to whether and under what conditions music is involved in the intricate network of cognition, emotional regulation, autonomic activity, behavioral and psychophysiological responses, and ultimately in people's mental health (Lin et al., 2011) has emerged from researchers in the field of psychology and neuroscience (Swaminathan and Schellenberg, 2018). While most of the researchers have expressed optimism about the benefits of music on cognition (Schellenberg, 2004; Slater et al., 2015; Tierney et al., 2015; Jaschke et al., 2018; Nan et al., 2018; Barbaroux et al., 2019), treatment of psychiatric disorders (Ho et al., 2003; Degé and Schwarzer, 2011; Chacón-Moscoso et al., 2016; Fang et al., 2017) as well as on people's overall wellbeing (Hsu and Lai, 2004), others have found this enthusiasm unjustified (Sala and Gobet, 2020), trying to explain and delineate some research quality failures that arise in the neuroplasticity and music field.

More specifically, in regards to the optimistic point of view, music-based intervention approaches, and practices favor research designs in this domain in relation to implementation and utilitarianism (Reybrouck and Eerola, 2017). That is, music can be experienced without necessitating a dedicated sensory organ, the ears, which are its main perceptual apparatus, and after vibrations through the peripheral nervous system, epithelium, and bones induce neuroplastic changes in the human brain. An example of this conclusion is that fetuses and deaf individuals can perceive and respond to music (Chorna et al., 2019). Likewise, the infants respond to the rhythmic dimensions of their mothers' speech and emotional tone because of humans' innate ability to engage with the “communicative musicality” of conversation (Lin et al., 2011). Also, researchers have found evidence of far-transfer effects related to “therapeutic” traits, or biological and cognitive paths of development (Miendlarzewska and Trost, 2013; Carter and Panisch, 2020). Typically, this means that a wide range of complex cognitive, emotional, behavioral, and psychophysiological responses can be adjusted through music and, as a result, the mental health of patients with various psychiatric disorders can be improved (Lin et al., 2011; Clift, 2012; Gustavson et al., 2021). Indeed, Gustavson et al. (2021) have claimed that therapies with active music participation, and structured and multiple sessions have significant positive effects on mood disorders (e.g., depression). Furthermore, Sanfilippo et al. (2021) have supported that passive music listening reduces anxiety and pain during labor, anxiety symptoms during pregnancy, and postnatal depression.

On the contrary, evidence of systematic reviews or meta-analyses has shown a pessimistic point of view, as described by few, concerning the music's usage and possible direct link to neuroplasticity. According to this literature, there is limited understanding and no clear evidence of how music, directly and indirectly, contributes to mental health (Lin et al., 2011; Gustavson et al., 2021). Also, the potential causational role of music in cognitive or academic development is very weak (Schellenberg, 2020) and conclusions of causation are precluded (Swaminathan and Schellenberg, 2018). Therefore, music does not reliably ameliorate psychiatric disorders (Lin et al., 2011) and enhance cognitive or academic skills, and there are non-pragmatic neuroplastic changes due to the inability to be reported as a causational link between music and neuroplastic development (Schellenberg, 2020, p. 430). As a result, positive correlational findings are probably due to confounding (e.g., individual differences) or unidentified variables (i.e., genetic, or demographic factors) that contribute to the confounding ones (Schellenberg, 2020, p. 431). Besides, far transfer effects of music on development and mental health appear to be an extremely rare occurrence, an over-optimistic and incorrect view, as they stem from a misinterpretation of the empirical data and possibly confirmation bias (Sala and Gobet, 2020). For example, Swaminathan et al. (2017) showed that the correlation between fluid intelligence and engagement in music in a sample of adults was mediated by innate personality factors (i.e., music aptitude) and not trained music skills. Based on this finding, the hypothesis that music training boosts cognition or academic skills cannot be supported. In another example of reviews investigating the effect of listening to music on anxiety symptoms during pregnancy, Sanfilippo et al. (2021) have argued that the positive correlational effect comes from the predominant use of self-reported measures and, as a consequence, it is not evident the exact mechanism through which music achieves the reduction of pregnant women's anxiety symptoms.

Moreover, the results of far transfer studies seem to be inconclusive or contradictory, due to non-specific or occasional methods used, absence of proper and structured classification of far transfer, lack of a structured understanding of music and musicality, as well as differences in neural activation during the processing of the tasks (Jaschke et al., 2013; Fang et al., 2017). An example that depicts the lack of uniformity in the test methods used is when comparing **two** different results of far transfer studies: on the **one** hand, Ho et al. (2003) did not show a positive transfer effect of music on visual memory, but on the other hand, Schellenberg (2004) did show a positive effect of music on intelligence using **two** different and non-specific IQ measures (Raven's standard matrices and general intelligence). Although both studies analyzed intelligence, Schellenberg (2004) may have a stronger effect sensitivity because of the generalized measures used. Also, as far as the contrasting far transfer results, some researchers claim that musicians may be at higher risk for mental health problems (Wesseldijk et al., 2019), but others suggest the opposite (Teorell et al., 2014; Johnson et al., 2017). As a result, there are no strong generalizations from their findings because of the variability in outcome measures and music intervention used from one study to another (Lin et al., 2011). In the same vein, immature field implementation of methods used and many times non-replicable findings are present in studies investigating the effect of music therapy on Alzheimer's Disease (AD) (Fang et al., 2017). For that reason, Fang et al. (2017) have argued that there are not as many clinical trials as possible with cohort, randomized, blinded, and rigorous methodological investigations for music's therapeutic effect on the topic of AD.

Additionally, meta-analyses that examined the causal link between musical and non-musical abilities reported skeptical results as this link is not clear-cut or, in the case of correlational studies, these associations are not always evident (Swaminathan and Schellenberg, 2018). Indeed, there are studies that their findings are more liable to yield a positive effect of music on cognition because the researchers adopt non-standard pedagogies (e.g., training in music-listening skills rather than teaching participants to sing or play an instrument) (Swaminathan and Schellenberg, 2018) (for example please see Degé and Schwarzer, 2011). Apart from that, firm conclusions referring to the protective effect of music on various psychiatric conditions are difficult to be drawn due to the mixed quality regulation shown in many studies (i.e., small sample sizes, lack of appropriate control groups, few interventions with multiple sessions, omitted necessary information such as inclusion/exclusion criteria regarding the intervention, lack of masking of interviewers during post-test, and randomization concealment) (Wesseldijk et al., 2019). As a result, it seems that some researchers cannot clarify how music leads to greater people's health and wellbeing (Gustavson et al., 2021).

## Discussing the ways forward

Many different neuroscientific and clinical studies have proven that music possesses a beneficial role in cognitive, behavioral, and emotional development (Miendlarzewska and Trost, 2013; Carter and Panisch, 2020), improving also the overall psychophysiological health of patients with various psychiatric disorders (Lin et al., 2011; Gustavson et al., 2021; Sanfilippo et al., 2021). Nonetheless, according to the aforementioned systematic reviews or meta-analyses (please see above for more details), various factors contribute to a blurred outcome due to non-unified research methods, inconsistent results, misinterpretation or distortion of empirical data, and low reproducibility and replicability of scientific findings, to name just a few. Whilst not all of these factors are necessarily problematic, we believe there is “room” for improvement and development in this research field. For this reason, we propose some state-of-the-art approaches to minimizing the frequency of commonly observed limitations, based on potential solutions that are observed to be universal in empirical research (Lin et al., 2011; Boutron and Ravaud, 2018; Brown et al., 2018; Jaschke et al., 2018; Sala and Gobet, 2020; Gustavson et al., 2021; Ganley et al., 2022), and likewise can be applied to this specific domain, too.

More specifically, as far as the limitation of non-unified research methods and heterogeneous results, research should perhaps be conducted through more longitudinal randomized controlled trials (RCTs) combined with both clinical and neuroscientific outcome measures, uniform methodological investigations (e.g., different kinds of control groups) and analysis of sub-groups of tasks proposed (Lin et al., 2011; Fang et al., 2017; Jaschke et al., 2018). Following this perspective, more reliable, accurate, and powerful results, and a consistent research protocol on far transfer from music to cognition/mental health studies can be produced. Besides, the interconnection between music, cognition, and mental health will be better disentangled, perhaps implementing new strategies for music therapy (Lin et al., 2011).

As far as the restriction of the results' ineffectiveness is concerned, some researchers suggest that a near transfer effect between music and personal development (e.g., self-esteem) could much easier be successful as it focuses on more technical and visible concepts (i.e., self-esteem) rather than far transfer one that represents a broader and less visible concept among music and cognitive development (e.g., music and cognitive development in mathematics) (Sala and Gobet, 2020). On the contrary, more demanding experimental intervention studies should be propitious on the limitations' resolution. That is, the use of larger samples, and improved reporting standards, combined with not only psychological, physiological, and neurochemical aspects, but also with genetic and environmental designs (e.g., longitudinal twin and family studies, for more details please see Purcell, 2002), neuroimaging methods (focusing on the reward's circuitry activation, and dynamic patterns of brain activity in health and disease due to music), and biobanks of electronic health record (EHRs) databases estimating music-mental health and other related health associations in large samples (Gustavson et al., 2021). As a consequence, the exact interactive mechanism across music and existing risk factors supporting mental health and overall wellbeing can be more easily explored by employing the above approaches.

Moreover, regarding the problem of data misinterpretation or distortion, we can suggest five different and essential solutions to resolve this limitation. Firstly, important information on the full experiment protocol, statistical analysis plan, or sequence of analytical choices and raw data for all designed research should be accessible, reducing the risk of confirmation bias (Boutron and Ravaud, 2018). Secondly, maximization of the effect-to-bias ratio in research through random group assignment, incorporation of blinding, and heterogeneity (if possible) into the design should be accomplished to enhance generalizability (Boutron and Ravaud, 2018). Thirdly, editors' journals should provide recommendations on how results should be interpreted (e.g., guidelines for the proper use and interpretation of statistical tools), how the conclusions should be reported, and how to avoid misrepresentation of data (Boutron and Ravaud, 2018). Fourth, researchers should not only raise evidence for systematic reviews or meta-analyses but also evaluate the quality of the papers themselves following standardized scoring systems (e.g., the “QualSyst” tool developed by Kmet et al., 2004) specialized for this kind of inquiry (Chacón-Moscoso et al., 2016). Lastly, additional training and tools for peer-reviewers and editors should be included in identifying misrepresentation in clinical research reports and outcome measures (Boutron and Ravaud, 2018).

Finally, in relation to the low reproducibility and replicability of scientific findings, in one way, universities should increase awareness of problems in research quality and teach their solutions by adding additional courses (e.g., courses on study design or statistical analysis) (Brown et al., 2018). In another way, proper funding and personnel for more pilot and feasibility studies should be important, minimizing many small, non-randomized studies with cross-sectional survey data, and a variety of non-validated questionnaires (Brown et al., 2018). Also, partnerships across researchers in academia and research organizations outside of academia (e.g., music industries) or private companies should result in the reassurance of data integrity and results in quality generated in this domain (Ganley et al., 2022).

## Conclusion

Due to the implementation of non-unified and blurred quality methodological standards, more justification and work are needed to explain music's possible causal link to neuroplasticity. Support of future studies may be apprehended through comparable and inter-connected related outcomes, allowing the presentation of new knowledge content and processes. Therefore, given the variability in published results in the specific field and the difficulty of interpreting these results, the development of stronger, more thorough, and more uniform research methods in quality is urgent (Jaschke et al., 2013) as has already been stated in the past (Chacón-Moscoso et al., 2016). Solutions mentioned previously such as RCTs, larger sample size, the 5 methods of overcoming data bias and error, and more extensive incorporation of study-validity and feasibility assessment would be our suggested manner of approaching such objectives. Thus, future studies of far transfer from music to mental health and cognitive development could be more reliable and accurate (Jaschke et al., 2013). However, these solutions should not be considered a panacea, as science will certainly continue to evolve in the future. Instead, they should be approached as “food-for-thought” for researchers, publishers, regulators, and stakeholders (e.g., funding agencies) to move forward.

## Author contributions

EPa and MA: conceptualization. EPa: methodology and supervision. MA and EPi: validation and resources. MA, EPi, and EPa: writing—original draft preparation and writing—review and editing. MA: project administration. All authors have read and agreed to the published version of the manuscript.

## Funding

The article publication charge for this work was funded by Oslo Metropolitan University.

## Conflict of interest

The authors declare that the research was conducted in the absence of any commercial or financial relationships that could be construed as a potential conflict of interest.

## Publisher's note

All claims expressed in this article are solely those of the authors and do not necessarily represent those of their affiliated organizations, or those of the publisher, the editors and the reviewers. Any product that may be evaluated in this article, or claim that may be made by its manufacturer, is not guaranteed or endorsed by the publisher.
